# Patterns of *Schistosoma haematobium *infection, impact of praziquantel treatment and re-infection after treatment in a cohort of schoolchildren from rural KwaZulu-Natal/South Africa

**DOI:** 10.1186/1471-2334-4-40

**Published:** 2004-10-07

**Authors:** Elmar Saathoff, Annette Olsen, Pascal Magnussen, Jane D Kvalsvig, Wilhelm Becker, Chris C Appleton

**Affiliations:** 1 Harvard School of Public Health, Department of Nutrition, 665 Huntington Avenue, Boston, MA 02115 USA; 2 Danish Bilharziasis Laboratory, Jaegersborg Allé 1D, DK-2920 Charlottenlund, Denmark; 3 Child, Youth and Family Development, Human Sciences Research Council, Private Bag X07, Dalbridge, 4014, South Africa; 4 Zoologisches Institut der Universität Hamburg, Martin-Luther-King-Platz 3, 20146 Hamburg, Germany; 5 School of Life and Environmental Sciences, University of KwaZulu-Natal, Durban 4041, South Africa

## Abstract

**Background:**

Schistosomiasis is one of the major health problems in tropical and sub-tropical countries, with school age children usually being the most affected group. In 1998 the Department of Health of the province of KwaZulu-Natal established a pilot programme for helminth control that aimed at regularly treating primary school children for schistosome and intestinal helminth infections. This article describes the baseline situation and the impact of treatment on *S. haematobium *infection in a cohort of schoolchildren attending grade 3 in a rural part of the province.

**Methods:**

Primary schoolchildren from Maputaland in northern KwaZulu-Natal were examined for *Schistosoma haematobium *infection, treated with praziquantel and re-examined four times over one year after treatment in order to assess the impact of treatment and patterns of infection and re-infection.

**Results:**

Praziquantel treatment was highly efficacious at three weeks after treatment when judged by egg reduction rate (95.3%) and cure rate of heavy infections (94.1%). The apparent overall cure rate three weeks after treatment (57.9%) was much lower but improved to 80.7% at 41 weeks after treatment. Re-infection with *S. haematobium *was low and appeared to be limited to the hot and rainy summer. Analysis of only one urine specimen per child considerably underestimated prevalence when compared to the analysis of two specimens, but both approaches provided similar estimates of the proportion of heavy infections and of average infection intensity in the population.

**Conclusion:**

According to WHO guidelines the high prevalence and intensity of *S. haematobium *infection necessitate regular treatment of schoolchildren in the area. The seasonal transmission pattern together with the slow pace of re-infection suggest that one treatment per year, applied after the end of summer, is sufficient to keep *S. haematobium *infection in the area at low levels.

## Background

Schistosomiasis is one of the major health problems in tropical and sub-tropical countries [1]. The schistosomiasis endemic area in South Africa is situated in the north-east and covers roughly one quarter of the country, with *Schistosoma haematobium *being the most common species [2]. In 1995 it was estimated that more than four million South Africans were infected with schistosomes [3].

Possible consequences of *S. haematobium *infection include haematuria, dysuria, nutritional deficiencies, lesions of the bladder, kidney failure, an elevated risk of bladder cancer and – in children – growth retardation. Accordingly the estimates for morbidity and mortality in affected populations are high [4-7]]. School age children usually present with the highest prevalence and intensity of *S. haematobium *infection [8]. However, negative health consequences are not limited to this group since high intensity infections can cause serious chronic disease long after initial infection [9]. Some studies also suggest that schistosomiasis may play a role as a risk factor for HIV infection and that helminth infections in general negatively affect the immune system of HIV infected persons [10,11].

In 1998 the Department of Health of the province of KwaZulu-Natal (KZN) in co-operation with the Department of Education established a pilot programme for helminth control that aimed at regularly treating primary school children for schistosome infections and intestinal helminth infections [12]. All children in participating schools were treated without prior screening of infection status. The rationale behind this and similar programmes in other countries is not to eliminate infection in a given area, but to keep infection intensities low in this vulnerable age group in order to prevent serious morbidity [13,14].

Our objectives were to describe the pattern of schistosome infection at baseline, to monitor the impact of treatment in our study population and to assess re-infection after treatment in order to develop recommendations for future control activities.

## Methods

### Study area, population and treatment

**Figure 1:**
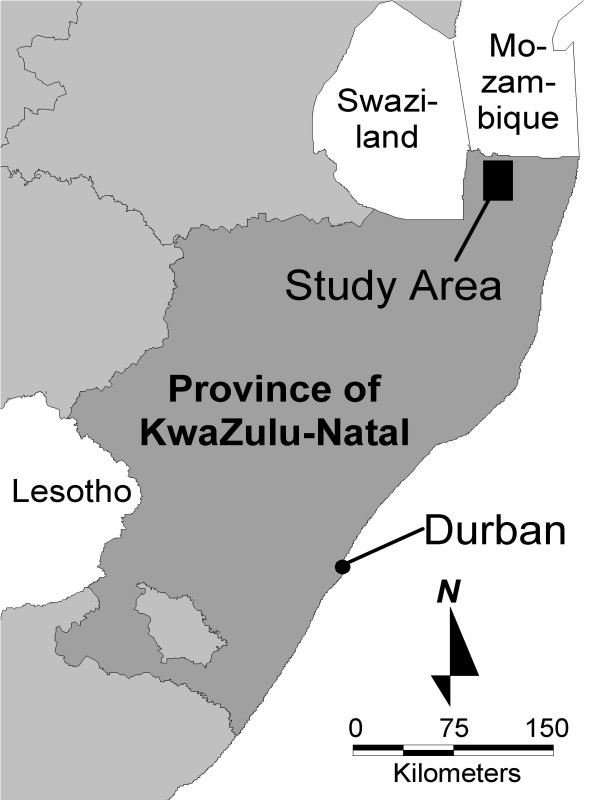
Location of the study area in northern KwaZulu-Natal.

**Figure 2:**
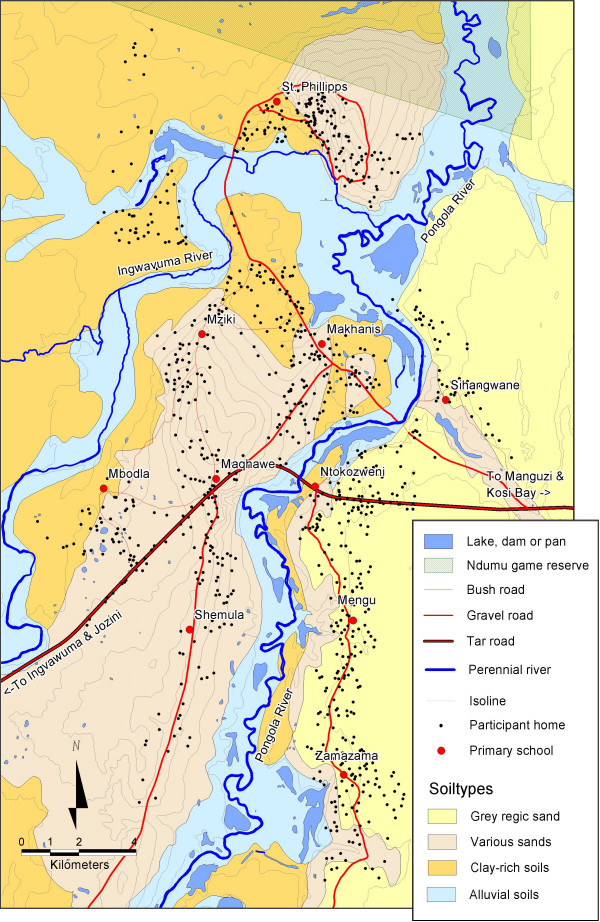
Map of the study area.

**Figure 3:**
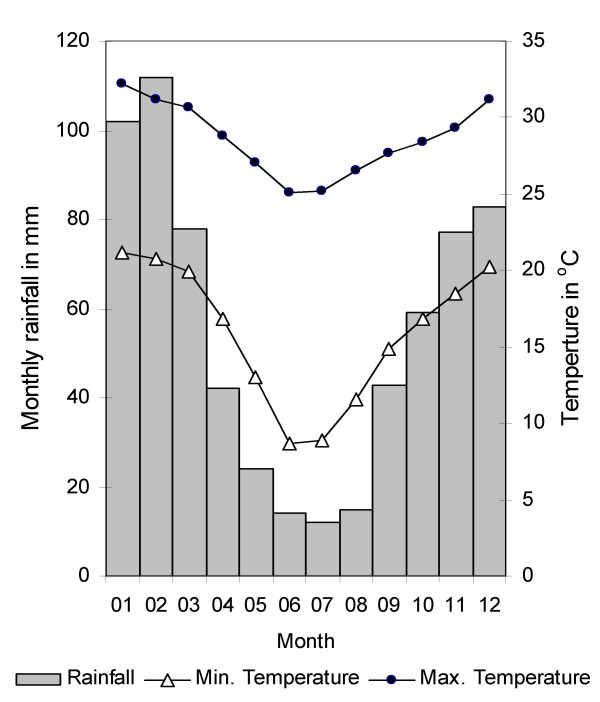
**Long-term monthly averages of rainfall and temperature in the area**. Data from 1966 to 1990 for Makatini research station, about 30 km south of the study area [36].

The study was conducted in central Ingwavuma district in northern KwaZulu-Natal (Figure 1). The study area covers approximately 28 × 16 km on both sides of the perennial Pongola River (Figure 2). Climate is tropical to subtropical (Figure 3) with a hot and wet summer (November – February) and a cooler and dry winter (June – August).

The study population (Table 1) was recruited from all ten primary schools in the area. It was limited to children who attended grade 3 at the start of the study in order to keep disturbances of the school routine to a minimum and because this grade should represent the infection situation in a primary school relatively well [15]. All grade 3 pupils were eligible for participation with one exception: during the baseline survey two out of five and one out of four grade 3 classes in two large schools had to be excluded due to logistic constraints. These classes, however, were included during treatment and successive surveys.

All pupils who provided a urine specimen during the pre-treatment survey were included in the analysis of infection patterns at baseline but only children who had been treated with praziquantel were included in the analysis of the post-treatment surveys. Of these, another 4.6% who reported having received additional treatment for schistosome infection while our study was ongoing were excluded from analysis. Otherwise only children who refused to participate or who were absent or unable to produce a specimen during each of our repeated visits were not included in the analysis of the respective surveys. They were included, however, for those surveys where they participated in order not to increase bias due to the likely difference in disease status between absentees and pupils who attended school [16].

Treatment in all primary schools in the entire district was carried out in April and May 1998 by school nursing teams from the two local hospitals as part of the first treatment campaign of a provincial helminth control programme. The study team assisted the nurses with treatment and also recorded those of the study population who were treated and those who were not. All consenting children from all grades were treated for schistosome infection with a single dose of 40 mg/kg praziquantel (Biltricide^®^, Bayer) without regard to infection status. In order to facilitate administration of the drug, the nurses had been provided with a dosing sheet that showed the correct dosage for different bodyweights. The weight of the children was determined using an ordinary bathroom scale. Because one Biltricide^® ^tablet containing 600 mg of praziquantel can be subdivided into four segments of 150 mg the required dose can be administered relatively accurately. The drug was administered with a glass of water after the children had eaten a peanut butter sandwich which was provided by the treatment team. Children were asked to swallow the tablets with some water in front of one of the team in order to monitor adherence. Because praziquantel is considered a safe drug and has been used extensively since its introduction in the early 80ies possible side effects of treatment were not monitored systematically [17].

Children were also treated for intestinal helminth infection with 400 mg albendazole (Zentel^®^, SmithKline Beecham) and albendazole treatment was repeated in October 1998 [18]. After the end of the study the participants were included in the normal treatment routine of the control programme.

### Ethical considerations

Ethical clearance was obtained from the Ethics Committee of the Faculty of Medicine of the University of Natal/Durban and the study was also approved by the Central Medical Ethics Committee in Denmark. Before the onset of the study, information meetings were held with the staff and parents of the schools in the study. At these meetings informed consent was obtained from the parents. Informed consent from the children was obtained directly before the first specimen collection.

### Specimen collection and processing

An initial survey in March 1998 to assess *S. haematobium *infection in the study population was followed by treatment for schistosome and intestinal helminth infections. Follow-up surveys to monitor loss of infection and re-infection were conducted at 3, 16, 41 and 53 weeks after treatment (Table 2).

Urine specimens were collected between 10:00 and 13:00 hours [13]. On our visits to the schools, pupils were provided with labelled 500 ml specimen containers and asked to provide a urine specimen. Each school was visited at least three times during each survey in order to include children who were absent or unable to deliver a specimen on the first occasion. Apart from the pre-treatment survey, where only one specimen was collected, an effort was made to obtain two urine specimens from each pupil. However, the results reported in this article are calculated using only the first specimen obtained. Otherwise the lower sensitivity of the pre-treatment survey would have invalidated comparisons with the follow-up surveys. The results of both obtained specimens are used only in Table 2 where we directly compare them to those of only one specimen.

Filled specimen containers were brought to the laboratory where filtration of a sub-sample of 10 ml was carried out on the same day [19]. Specimens of less than 10 ml were measured before filtration and the number of eggs per 10 ml calculated. Before microscopy, eggs were stained using 50% Lugol's iodine saline solution [20] and then counted by 3 microscopists in order to obtain an indirect measurement of infection intensity. These counts did not differentiate between viable and non-viable eggs. Repeat counts by different microscopists were done on a sub sample of about 5% of the slides for quality control purposes. These counts revealed no bigger discrepancies. Infection intensities are expressed as eggs per centilitre (EPC, 1 centilitre = 10 ml).

### Statistics

Data were double entered, the duplicates compared and corrected for data entry errors. Statistical analysis was carried out in Stata 7 for Windows [21].

In order to reduce the influence of extreme outliers, geometric means were preferred to arithmetic means to summarise population infection intensity (Table 1 and Table 2). Uninfected children were included by adding 1 to all egg counts before log transformation and subtracting it again after re-transformation. However, when comparing intensity, non-parametric statistics were used because even the log transformed egg counts were still far from being normally distributed. Cure rates (CR) and egg reduction rates (ERR) were calculated using the formulae below [15]:


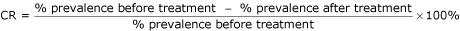



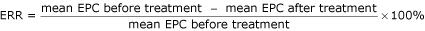


## Results

### Infection patterns at baseline

**Figure 4:**
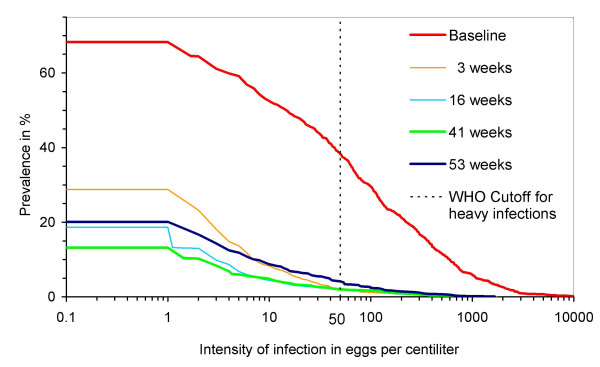
***Schistosoma haematobium *infection at baseline and at various periods after treatment**. Prevalence of infections >= any intensity threshold of interest can be read from the percentage scale. The intersection of each graph with the y-axis corresponds to the total prevalence. For the number of participants in each survey see Table 2.

In the pre-treatment survey 68% of the study population were found infected with *S. haematobium *and 38% (= 56% of the infected children) had egg counts of 50 or more EPC, the WHO [4] threshold for heavy intensity infections (Figure 4). As shown in Table 1, differences between sexes regarding prevalence and intensity of *S. haematobium *infection were moderate.

**Figure 5:**
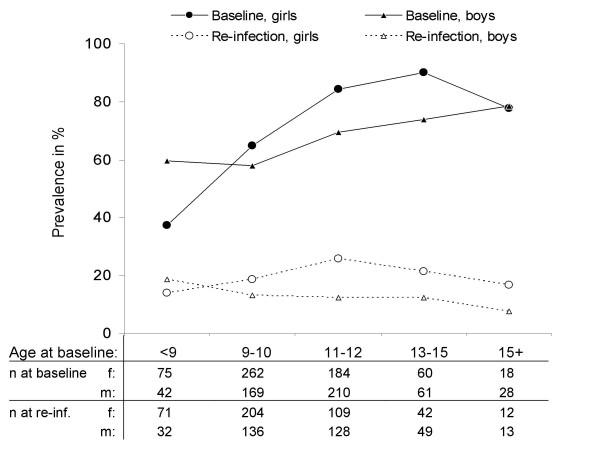
**Prevalence of *Schistosoma haematobium *infection before treatment (baseline) and after 53 weeks of re-infection**. Re-infection data only include children who were found egg-negative in at least one of the surveys at 3, 16 or 41 weeks after treatment. The table below the graph shows the number of participants in each age/sex stratum.

Figure 5 demonstrates that age patterns of infection at baseline differed considerably between sexes. *S. haematobium *prevalence of boys slowly increased from 60% in the youngest to 79% in the oldest age group whereas the youngest girls had a much lower prevalence (37%) but the increase with age was steeper.

### Treatment

Of the 1109 children who participated in the baseline survey only 852 (76.8%) were treated with praziquantel. 228 children (20.6%) did not receive treatment either because they refused to be treated (7 children) or because they were absent on treatment day (221) and for 29 children (2.6%) it is unclear whether or not they were treated. Differences between the treated and untreated groups (excluding children of unclear treatment status) regarding sex, age, infection status and infection intensity were small and not statistically significant.

*S. haematobium *infection status, CR and ERR over 53 weeks after treatment are summarised in Table 2. Infection intensity measured as the geometric mean EPC had decreased by more than 95% as soon as three weeks after treatment and over the following months a further small decrease is documented until 41 weeks after treatment. The pattern for prevalence and CR of heavy intensity infections is very similar to this. Total prevalence shows the same trend over time, but in contrast exhibits considerably smaller decreases than the other two measures.

In Table 2 we also report the results that were obtained when using both specimens that were collected in the post-treatment surveys. When comparing the different approaches it is obvious that – as expected – the use of only one sample considerably underestimates the total prevalence in this population, but that estimates for prevalence of heavy infections and geometric mean intensity are quite similar.

### Re-infection

No discernible re-infection took place between 3 and 41 weeks after treatment (Table 2 and Figure 4) whereas the increase between 41 and 53 weeks after treatment was substantial (two-sided *p *< 0.001 for both prevalence and intensity using the sign test for equality of paired observations [22]).

The group at risk of re-infection (Figure 5) was defined as those children who were found uninfected in at least one of the three surveys at 3, 16 or 41 weeks after treatment (n = 796). Fifty-three weeks after treatment 16.8% of these children were found to be re-infected with *S. haematobium*. The geometric mean EPC including uninfected children of 0.44 was still far below the corresponding figure before treatment (16.08) and this was also true for the geometric mean EPC when excluding uninfected children (58.2 before treatment and 6.6 at 53 weeks after treatment).

Figure 5 shows that the age pattern of re-infection in the study population also differed between sexes: it continuously decreased with age for boys but not for girls where re-infection reached a peak in the group that had been 11 to 12 years old at the time of the baseline survey and that was about 12 to 13 years old at the end of the study.

## Discussion

### Infection patterns at baseline

The results of our baseline survey are in agreement with a survey conducted in the area about 20 years earlier. Schutte et al. [23] found prevalences of between 55% and 92% in the four surveyed schools that were situated in our study area. The high total prevalence and the large proportion of high intensity infections that we found indicate that according to WHO criteria regular treatment of schoolchildren in the area is indeed necessary [4]. The different age patterns for prevalence of *S. haematobium *infection in girls and boys might hint at different water contact patterns.

### Treatment

The proportion of children treated in our study can not be regarded as representative for the control programme in general, because study participants were more informed about it than their schoolmates. However, the fact that only about three quarters of the children were treated is very disappointing. Although only 3 children did not consent to participate in our study, and only 7 of those who consented openly refused to be treated absenteeism was unusually high during the first round of treatment. This improved greatly in the second treatment half a year later, when – according to the opinion of school staff – pupils and their parents had realised that treatment was beneficial and had only mild and transient side effects. Unfortunately this second round of treatment did only include albendazole but not praziquantel because the schedule of the provincial treatment programme only provided one treatment for schistosome infection per year.

Praziquantel treatment resulted in drastic reductions of infection intensity and prevalence of heavy infections as soon as three weeks after treatment, which is in accordance with the literature [24]. The reduction in overall prevalence of less than 60% at three weeks after treatment is however unsatisfactory, even though it improved to about 80% at 41 weeks after treatment. The explanations that the treatment did not work or that its effect was delayed can be excluded because of the high ERR. It seems more likely that the relatively high post-treatment prevalences are not an indication of a high proportion of active infections after treatment, but that they are caused by "old" and mostly non-infective eggs, laid before treatment, that were trapped somewhere in the tissue and are slowly finding their way to the lumen of the bladder [25,26]. Unfortunately our laboratory examination did not differentiate between viable and non-viable eggs, but the data presented in Figure 4 are consistent with the above explanation because very little change is visible between 3 and 41 weeks after treatment with regard to infections of more than 50 EPC. During this period prevalence decreased almost exclusively in the low intensity range. If many active infections (= egg laying schistosomes) had been lost, this should also have had an impact on infections of higher intensity.

The high variability of repeated *S. haematobium *egg counts [27] renders single egg counts a less than optimal tool for estimating total prevalence and for identifying the infection status of individuals. Our results however show that estimates of the proportion of heavy infections and of population infection intensity are similar to those obtained when examining two specimens. The examination of three or more specimens per child would most certainly have led to even higher estimates of total prevalence but we doubt that it would have changed the other two estimates considerably.

All this indicates that the reporting of measures of infection intensity is not only important because they are a better indicator of population morbidity than prevalence [8,15], but that intensity is also a more reliable marker of treatment success defined as the removal of egg-laying worms. This is especially important when relying on single egg counts to assess the effectiveness of the intervention which is usually the case in treatment programmes and larger field studies [28].

### Re-infection

Because of the slow decrease in total prevalence after treatment it did not seem appropriate to restrict analysis of re-infection to those children who were egg-negative three weeks after treatment. According to our above reasoning this would have excluded a number of children who had been treated successfully but were still excreting old eggs. On the other hand we did not want to restrict the analysis to those children who were found egg-negative at 41 weeks after treatment. Even though this was the survey where we found the lowest prevalence, we might have excluded children who had been treated successfully, but had become re-infected again before this survey. Therefore we included all children who were found egg-negative at either 3, 16 or 41 weeks after treatment into the group at risk of re-infection. We are, however, aware that this definition is likely to include some uncured children who were still harbouring low level infections.

Our data indicate that *S. haematobium *transmission occurred mainly during the hot and humid summer. According to the literature, *S. haematobium *has a pre-patent period (infection to egg-excretion by the host) of about eight to ten weeks [29,30]. Thus the surveys at 41 weeks and at 53 weeks after treatment should approximately reflect transmission from early May (treatment) to early December and from early December to late February respectively. The former period covers the South-African, winter, spring and only a short part of the summer whereas the latter covers most of the hottest and wettest part of the year (Figure 3), conditions which favour *S. haematobium *transmission and the development of their *Bulinus globosus *snail hosts [31,32]. Moreover, children go swimming more frequently because of the hot weather and the long summer holidays in December and January give them ample time to do so.

Seasonality of *S. haematobium *transmission is well documented for the highveld region of Zimbabwe[33] with patterns rather similar to the ones found here, which seem to be caused by seasonal variation in snail populations as well as human water contact patterns [34]. A study in southern Natal found that recreational activities accounted for most of the water contact and – unlike household related water contacts – showed strong seasonal variation [35].

## Conclusions

Our study shows that according to WHO guidelines [4] the high prevalence and intensity of *S. haematobium *infection in the area indeed necessitate regular treatment of schoolchildren, that praziquantel treatment is highly efficacious in reducing the proportion of moderate and heavy infections and that one treatment per year after the end of summer is sufficient to keep infection intensities at low levels. Because levels of *S. haematobium *infection in the study area seem to be among the highest in the country [23] the latter would probably also apply to other parts of South Africa. The slow pace of re-infection might suggest that it could even be sufficient to only treat every two years, but this would need to be verified in a separate study that follows treated children over this period.

Because we would not want to discourage future attempts to control schistosomiasis in the region and elsewhere we would finally like to stress that the above described money and time consuming intensive examinations are only required when doing research but that for a control programme a minimum of surveillance is sufficient [4].

## Competing interests

The authors declare that they have no competing interests.

## Authors' contributions

ES conceived of the study and designed it together with AO, PM, JDK and CCA. ES conducted the field work with contributions from AO, JDK, CCA and WB, did the statistical analysis and drafted the manuscript. All authors contributed to the final version of the manuscript and read and approved it.

## Pre-publication history

The pre-publication history for this paper can be accessed here:



## Figures and Tables

**Table 1:** Age and sex of the study population and prevalence and intensity of *Schistosoma haematobium *infection at baseline

n =	Males 510	Females 599	Male/Female Ratio	95%CI or *P*-value
Median age (inter-quartile range)	11.3 (9.8–12.2)	10.7 (9.7–11.5)	-	-
Prevalence (%)	65.9	70.3	0.937	0.864–1.017
Age adjusted prevalence ratio*	-	-	0.896	0.828–0.969
Prevalence of infections >= 50EPC in %	36.7	39.6	0.927	0.797–1.078
Geometric mean EPC incl. uninfected	14.3	17.8	0.805	*p *= 0.151^†^

*Mantel-Haenszel age adjusted male/female prevalence ratio

^†^Two-sided *P*-value of two samples Wilcoxon ranksum test with ties

**Table 2:** *Schistosoma haematobium *prevalence and infection intensity, cure rates and egg reduction rates at various periods after treatment with 40 mg/kg praziquantel

Weeks since treatment	Pre-treatment	3	16	41	53
Time of survey	Mar '98	May/Jun	Aug	Feb '99	Apr/May

n =	1109	977	922	848	825

First specimen only*					
Prevalence (%)	68.3	28.8	18.7	13.2	20.1
Cure rate (%)	-	57.9	72.7	80.7	70.5
Prevalence of infections >= 50 EPC (%)	38.2	2.3	2.1	1.9	4.1
Cure rate of infections >= 50 EPC (%)	-	94.1	94.6	95.1	89.2
Geometric mean EPC incl. uninfected	16.1	0.8	0.4	0.3	0.6
Egg reduction rate (%)	-	95.3	97.5	97.9	96.0
Both specimens^†^					
Prevalence (%)	-	39.9	29.3	17.6	28.7
Prevalence of infections >= 50 EPC (%)	-	2.5	2.1	1.9	5.0
Geometric mean EPC incl. uninfected	-	0.9	0.5	0.3	0.6

*Results obtained when utilising only the first of two specimens that were collected

^†^Results obtained when utilising both specimens that were collected
